# Epidemiologia e Mortalidade da Doença Valvar Cardíaca em Adultos Brasileiros: Achados da Coorte ELSA-Brasil

**DOI:** 10.36660/abc.20260060

**Published:** 2026-06-26

**Authors:** Guilia Bevilacqua Schmitz, Eduardo Gatti Pianca, Maria Do Carmo Pereira Nunes, Bruce Bartholow Duncan, Murilo Foppa, Angela Barreto Santiago Santos

**Affiliations:** 1 Faculdade de Medicina Universidade Federal do Rio Grande do Sul Porto Alegre RS Brasil Pós-Graduação em Cardiologia e Ciências Cardiovasculares – Faculdade de Medicina – Universidade Federal do Rio Grande do Sul, Porto Alegre, RS – Brasil; 2 Hospital de Clínicas de Porto Alegre Porto Alegre RS Brasil Serviço de Cardiologia, Hospital de Clínicas de Porto Alegre, Porto Alegre, RS – Brasil; 3 Faculdade de Medicina Hospital das Clínicas Universidade Federal de Minas Gerais Belo Horizonte MG Brasil Faculdade de Medicina, Hospital das Clínicas, Universidade Federal de Minas Gerais, Belo Horizonte, MG – Brasil; 4 Universidade Federal do Rio Grande do Sul Porto Alegre RS Brasil Pós-Graduação em Epidemiologia, Universidade Federal do Rio Grande do Sul, Porto Alegre, RS – Brasil

**Keywords:** Doenças das Valvas Cardíacas, Epidemiologia, Mortalidade, Adulto

## Abstract

**Fundamento:**

A maior parte das evidências atuais sobre doença valvar cardíaca (DVC) vem de países de alta renda. O acesso ao tratamento e o impacto da DVC na qualidade de vida podem variar substancialmente entre países, principalmente devido a diferenças socioeconômicas.

**Objetivo:**

Este estudo teve como objetivo estimar a carga de DVC e sua associação com mortalidade em um país de renda média.

**Métodos:**

Incluímos participantes da coorte ELSA-Brasil que realizaram ecocardiograma transtorácico na linha de base (3.267 participantes; 1.539 homens; idade média de 60,6 ± 8,8 anos) em seis estados brasileiros. Os participantes foram categorizados em três grupos – sem DVC, DVC mínima/leve e DVC moderada/grave – e suas características e fatores associados foram analisados. Valores de p < 0,05 foram considerados estatisticamente significativos.

**Resultados:**

Entre todos os participantes, 2,4% apresentavam DVC moderada/grave (incluindo sete participantes com prótese valvar) e 43,6% apresentavam DVC mínima/leve. A DVC moderada/grave mais prevalente foi a regurgitação mitral, seguida pelas regurgitações aórtica e tricúspide, com 56 participantes (86%) apresentando regurgitação valvar isolada. A DVC moderada/grave foi mais comum em indivíduos mais velhos e esteve associada a maior prevalência da maioria das comorbidades cardiovasculares, porém a menores taxas de obesidade. Também se associou a maior remodelamento cardíaco e pior função sistólica, com variações fenotípicas conforme a valva acometida. A DVC moderada/grave esteve associada a maior mortalidade em 10 anos (HR: 3,7; IC 95% [2,17–6,31]), independentemente de idade, sexo e hipertensão.

**Conclusão:**

A DVC é comum entre adultos em uma coorte comunitária de um país de renda média e aumenta com a idade. Participantes com DVC moderada/grave apresentaram maior carga de comorbidades, remodelamento cardíaco mais acentuado, pior função sistólica e redução substancial da sobrevida em longo prazo.

## Graphical abstract

**Figura Central:**
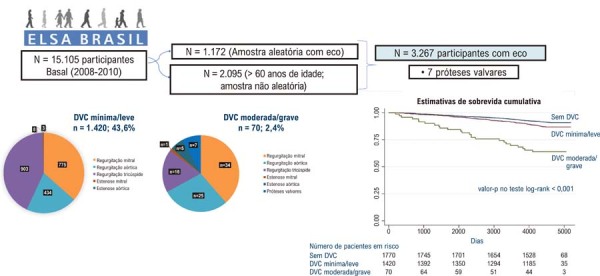
: Epidemiologia e Mortalidade da Doença Valvar Cardíaca em Adultos Brasileiros: Achados da Coorte ELSA-Brasil

## Introdução

A doença valvar cardíaca (DVC) é uma condição cardíaca prevalente, associada ao aumento da morbidade e mortalidade,^1-3^ e sua prevalência aumenta com a idade.^4-6^ Embora a maioria dos pacientes apresente DVC leve e assintomática, aqueles com doença moderada ou grave podem desenvolver complicações significativas, incluindo insuficiência cardíaca e morte.^2,3^ Além disso, o impacto da DVC na qualidade de vida e no acesso ao tratamento varia de acordo com o contexto socioeconômico de cada país.

Diversos fatores já foram associados a diferentes tipos de DVC. A regurgitação mitral está relacionada à idade avançada, sexo feminino, hipertensão, redução da fração de ejeção (FE) do ventrículo esquerdo (VE) e menor índice de massa corporal (IMC).^2,5,7^ A regurgitação tricúspide pode estar associada à idade avançada, sexo feminino, IMC reduzido, consumo de álcool, níveis mais altos de HDL-colesterol, frequência cardíaca elevada, disfunção do VE, doença cardíaca do lado esquerdo e aumento do átrio esquerdo.^6-8^ Já a regurgitação aórtica está principalmente associada à idade avançada, sexo masculino, pressão arterial sistólica elevada, dilatação da raiz aórtica e aumento do índice de massa ventricular esquerda.

A maioria dos dados disponíveis sobre DVC vem de países de alta renda, e evidências populacionais da América Latina continuam escassas, dificultando a estimativa precisa da carga de DVC nessa região. A coorte ELSA-Brasil oferece uma oportunidade única para obter estimativas confiáveis da prevalência e dos desfechos da DVC, além de avaliar o impacto da transição epidemiológica em curso em um país de renda média. Portanto, o presente estudo teve como objetivo investigar: 1) a prevalência de DVC em uma população adulta brasileira de base comunitária; 2) os determinantes clínicos e demográficos da DVC; 3) o fenótipo ecocardiográfico de cada DVC moderada/grave; e 4) o impacto da DVC moderada/grave na mortalidade em longo prazo.

## Materiais e Métodos

### População do estudo

O Estudo Longitudinal de Saúde do Adulto (ELSA-Brasil) é um estudo epidemiológico prospectivo desenvolvido para investigar doenças cardiovasculares e diabetes em 15.105 servidores públicos, homens e mulheres, de universidades ou instituições de pesquisa localizadas em seis cidades brasileiras (São Paulo, Rio de Janeiro, Belo Horizonte, Vitória, Salvador e Porto Alegre). Todos os funcionários ativos ou aposentados, com idades entre 35 e 74 anos, eram elegíveis para participar. Os detalhes do estudo — incluindo desenho, critérios de elegibilidade, recrutamento e avaliações — foram descritos previamente.^9,10^

Uma subamostra aleatória da coorte foi definida *a priori* para análises do tipo caso-coorte. Essa amostra aleatória (n = 1.543) correspondeu a 10% do total da coorte, selecionada utilizando os mesmos critérios de estratificação aplicados à amostra completa (sexo, faixa etária e categoria ocupacional). Um número aleatório gerado por computador foi atribuído a cada participante, e os 10% menores números dentro de cada estrato foram selecionados. Dessa amostra aleatória, ecocardiogramas transtorácicos (ETT) foram obtidos durante a primeira visita do estudo em 1.172 participantes.

**Figura 1:**
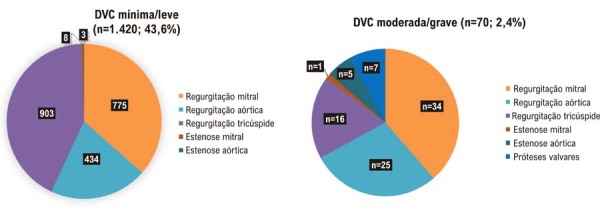
– Prevalência de doença valvar cardíaca (DVC) na amostra.

Para a presente análise, incluímos todos os participantes da coorte que realizaram ETT basal (agosto de 2008 a dezembro de 2010), priorizando todos os indivíduos com 60 anos ou mais (N = 2.095), bem como a subamostra aleatória predefinida correspondente a 10% de toda a coorte (N = 1.172) (Material Suplementar – Figura 1).

Os dados de mortalidade por todas as causas até dezembro de 2022 foram coletados por meio de acompanhamento telefônico anual e confirmados por revisão de prontuários médicos, certidões de óbito e vinculação a bases de dados nacionais, como o Sistema de Informações sobre Mortalidade.

O estudo ELSA-Brasil está em conformidade com os princípios da Declaração de Helsinque e foi aprovado pelos comitês de ética em pesquisa das instituições participantes. Todos os participantes forneceram consentimento informado por escrito.

### Ecocardiografia

Todos os ecocardiogramas transtorácicos foram realizados durante a primeira visita do estudo. As imagens foram adquiridas utilizando aparelhos de ecocardiografia configurados de forma idêntica (Aplio XG, Toshiba, Japão), equipados com transdutor setorial de 2,5 MHz, seguindo protocolos padronizados. Os exames foram gravados em formato digital e transferidos para o Centro de Leitura de Ecocardiografia do ELSA-Brasil, em Porto Alegre, Brasil. Parâmetros ecocardiográficos e Doppler padrão foram analisados em uma estação de trabalho offline (ComPACS 10.5; Medimatic SrL, Itália). Todas as medidas foram realizadas em triplicata, de acordo com as recomendações da *American Society of Echocardiography*,^11^ e incluíram diâmetros e volumes do ventrículo esquerdo (VE), espessura das paredes do VE, massa ventricular esquerda, fração de ejeção do VE e volume e diâmetro do átrio esquerdo (AE).

A gravidade da DVC foi inicialmente sinalizada pelo ecocardiografista treinado durante a aquisição das imagens, e imagens adicionais além do protocolo pré-definido foram obtidas a critério do operador para melhor caracterização da DVC, incluindo avaliação de prolapso da valva mitral e morfologia de valva aórtica bicúspide. Todas as imagens foram posteriormente revisadas no centro de leitura (independentemente de terem sido sinalizadas inicialmente) e avaliadas qualitativamente com base nos achados de Doppler colorido e Doppler espectral, seguindo critérios aplicados em estudos populacionais anteriores.^12^

Além disso, a definição de estenose aórtica (EA) foi baseada na velocidade sistólica anterógrada através da valva aórtica, enquanto a classificação da estenose mitral (EM) foi baseada no gradiente médio transmitral.

O protocolo do estudo teve como objetivo específico identificar prolapso da valva mitral, definido como espessura máxima do folheto >5 mm e deslocamento sistólico >2 mm além do plano do anel na visão paraesternal, bem como a morfologia de valva aórtica bicúspide, caracterizada por coaptação excêntrica dos folhetos, presença de dois folhetos durante a sístole e orifício sistólico elíptico. A hipertensão pulmonar foi definida como gradiente átrio direito–ventrículo direito (AD–VD) >25 mmHg.

### Análise estatística

Classificamos os participantes em três grupos: sem DVC, DVC mínima/leve e DVC moderada/grave. As variáveis contínuas são apresentadas como mediana e intervalo interquartil e foram comparadas utilizando o teste de Kruskal–Wallis. Quando o valor de p global foi <0,05, comparações pós-hoc pareadas foram realizadas usando o teste de Dunn com correção de Bonferroni para múltiplas comparações. As variáveis categóricas são apresentadas como contagens e proporções e foram comparadas utilizando o teste exato de Fisher, seguido por testes exatos de Fisher pareados quando o valor de p global foi <0,05, com valores de p ajustados pelo método de Bonferroni.

Cada doença valvar foi analisada ao longo das décadas de idade utilizando o teste não paramétrico de tendência de Cuzick (nptrend). Os correlatos relevantes das DVC moderadas/graves mais prevalentes – regurgitação mitral (RM), regurgitação aórtica (RA) e regurgitação tricúspide (RT) – foram inicialmente explorados em análises univariadas, seguidas por modelos de regressão logística multivariada incluindo covariáveis selecionadas a priori com base na literatura estabelecida, representando fatores de risco clinicamente distintos para doença valvar (idade, sexo, raça negra, tabagismo, obesidade, hipertensão e diabetes). Além disso, avaliamos os principais parâmetros ecocardiográficos associados a fenótipos específicos de DVC (volume diastólico final do VE, volume sistólico final do VE, espessura relativa da parede do VE, massa ventricular esquerda, fração de ejeção do VE, índice de volume do AE e hipertensão pulmonar), utilizando modelos de regressão logística ajustados por idade e sexo.

A sobrevida cumulativa foi estimada pelo método de Kaplan–Meier, e as curvas de sobrevida foram comparadas pelo teste de log-rank. Participantes com próteses valvares foram incluídos na prevalência geral de DVC moderada/grave, mas excluídos das análises subsequentes de caracterização devido às alterações na história natural da DVC.

Todas as análises estatísticas foram realizadas utilizando o pacote STATA (versão 12; StataCorp, College Station, Texas). Todos os testes foram bicaudais, e valores de p <0,05 foram considerados estatisticamente significativos.

## Resultados

Nossa amostra foi composta por 3.267 participantes (1.539 homens; idade média de 60,6 ± 8,8 anos; 56% autodeclarados brancos) provenientes de seis diferentes estados do Brasil (15% do Nordeste, 63% do Sudeste e 22% do Sul). A prevalência de hipertensão foi de 51,0%; diabetes 23,4%; sobrepeso 42,8%; obesidade 22,2%; doença arterial coronariana 6,5%; e tabagismo 10,2% na população geral.

Entre todos os participantes, 2,4% apresentavam DVC moderada/grave (incluindo 7 participantes com próteses valvares) e 43,6% apresentavam DVC mínima/leve (Figura 1 e Ilustração Central). A DVC moderada/grave mais prevalente foi a regurgitação mitral, seguida por regurgitação aórtica e tricúspide. Entre aqueles com DVC moderada/grave, 56 (86%) apresentavam regurgitação valvar isolada; 8 (12,5%) tinham doença mista envolvendo duas valvas regurgitantes; e 1 (1,5%) apresentava doença mista envolvendo três valvas regurgitantes. Estenoses moderadas/graves estavam presentes em 6 participantes (8,5% dos casos de DVC moderada/grave). Além disso, a prevalência de prolapso da valva mitral foi de 1,2% e de valva aórtica bicúspide de 0,5%, sem diferenças significativas entre os sexos.

Participantes com mais de 55 anos apresentaram maior prevalência tanto de DVC mínima/leve quanto de DVC moderada/grave em comparação com indivíduos mais jovens. Apenas quatro casos de DVC moderada/grave foram identificados entre participantes com menos de 55 anos, correspondendo a uma prevalência inferior a 1% (Material Suplementar – Tabela 1).

Os participantes com DVC moderada/grave eram mais velhos e apresentavam maior prevalência de hipertensão, doença arterial coronariana, insuficiência cardíaca, tabagismo e *flutter*/fibrilação atrial, além de menor prevalência de obesidade e síndrome metabólica (Tabela 1).

**Tabela 1:** – Características demográficas basais e parâmetros ecocardiográficos dos participantes do ELSA-Brasil que realizaram ecocardiograma na linha de base, estratificados pela gravidade da doença valvar cardíaca

	Sem DVC (n=1.770)	DVC mínima/leve (n=1.420)	DVC moderada/grave VHD (n=70)	Valor p
**Idade (anos)**	61 (51–65)	64 (60-68)	65 (61-71)*^†^	<0,01
**Sexo**				
Feminino	909 (51,3)	779 (54,9)	38 (54,3)	0,13
Masculino	861(48,6)	641 (45,14)	32 (45,7)	0,13
**Categoria de cor/raça autodeclarada**				0,005
Branca	993 (55,0)	783 (43,4)	29 (1,6)	
Preta	243 (48,9)	231 (46,6)	22 (4,4)	
Parda	442 (55,0)	346 (43,0)	16 (2,0)	
Amarela	44 (55,0)	35 (43,8)	1 (1,2)	
Indígena	26 (68,4)	11 (29,0)	1 (2,6)	
**Centros brasileiros**				<0,01
Bahia	218(44,1)	261(52,8)	15 (3,1)	
Espírito Santo	158(56,4)	119(42,5)	2 (1,1)	
Minas Gerais	390(47,5)	411(50,1)	20 (2,4)	
Rio de Janeiro	238(75,8)	73(23,2)	3 (1,0)	
São Paulo	377(58,9)	246(38,4)	17 (2,7)	
Rio Grande do Sul	389(54,7)	310(43,6)	12 (1,7)	
**PAS (mmHg)**	122,5 (112,5-134)	125 (114-139)	132 (118-142)*	<0,01
**PAD (mmHg)**	76 (69,5-83)	75,5 (69-82,5)	71,8 (65,5-80)*^†^	<0,01
**IMC (Kg/m^2^)**	26,9 (24,3-29,9)	26 (23,6-28,9)	25,4 (23,3-28,3)*	<0,01
**Sobrepeso**	769 (43,5)	597 (42)	28 (40)	0,66
**Obesidade**	437 (24,7)	275 (19,4)	10 (14,3)*	<0,01
**Hipertensão**	852 (48,2)	760 (53,6)	49 (70)*†	<0,01
**Diabetes**	408 (23,1)	338 (23,8)	15 (21,4)	0,85
**Síndrome metabólica**	944 (53,6)	710 (50,1)	29 (41,4)	0,03
**Acidente vascular cerebral**	33 (1,9)	29 (2,0)	3 (4,3)	0,27
**DAC**	94 (5,5)	101 (7,3)	12 (17,4)*^†^	<0,01
**Insuficiência cardíaca**	36 (2)	51 (3,6)	14 (20)*^†^	<0,01
**Tabagismo**	220 (12,4)	107 (7,5)	6 (8,6)	<0,01
**Flutter/fibrilação atrial**	6 (0,3)	17 (1,2)	4 (5,7)*^†^	<0,01
**Diâmetro diastólico do VE, mm**	45 (42-48)	45 (41-48)	50 (45-56)*^†^	<0,01
**Diâmetro sistólico do VE, mm**	28 (25-30)	28 (25-31)	31 (29-40)*^†^	<0,01
**Espessura do septo**	10 (9-11)	10 (9-11)	10 (9-12)*	<0,01
**Espessura da parede posterior, mm**	9 (8-10)	9 (8-10)	9 (8-10)	0,5
**Espessura relativa da Parede do VE**	0,42 (0,38-0,47)	0,42 (0,38-0,48)	0,38 (0,34-0,44)*^†^	<0,01
**Massa do VE, g**	138,7 (114,7-167,5)	140 (116,8-170,8)	177,8 (131,6-242,2)*^†^	<0,01
**Volume diastólico do VE, mL**	89,1 (77,9-101,6)	89,3 (77,3-103,9)	112,1 (92,7-142,4)*^†^	<0,01
**Volume sistólico do VE, mL**	28,5 (23,7-34,2)	28,5 (23,3-34,7)	36,4 (30,5-59,2)*^†^	<0,01
**Fração de ejeção do VE, %**	67,9 (62,5-72,2)	68,2 (62,8-72,9)	63,5 (52,3-68,6)*^†^	<0,01
**Volume do AE, mL**	45 (36,6-54,1)	47,1 (38,3-58,3)	59,8 (43,8-76,6)*^†^	<0,01
**Índice de volume do AE, mL/m^2^**	25,1 (20,8- 29,9)	27,2 (22,5- 32,6)	34,1 (25,8-45,5)*^†^	<0,01
**Hipertensão pulmonar**	25 (1,4)	291 (20,5)	18 (25,7)*	<0,01

Os valores são apresentados como mediana e intervalo interquartil, ou n (%). O valor de p foi calculado pelo teste de Kruskal–Wallis (análise pós-hoc usando o teste de Dunn) ou χ^2^ (análise pós-hoc usando o teste χ^2^), com correção de Bonferroni para múltiplas comparações quando o p global < 0,05. \ *p < 0,05 vs. sem DVC; ^†^p < 0,05 vs. DVC mínima/leve. DAC: doença arterial coronariana; IMC: índice de massa corporal; PAD: pressão arterial diastólica; PAS: pressão arterial sistólica; VE: ventrículo esquerdo; AE: átrio esquerdo.

As análises univariadas dos determinantes clínicos e demográficos de RM, RA e RT moderadas/graves estão apresentadas nas Tabelas Suplementares. A Tabela 2 resume os resultados das análises de regressão logística multivariada.

**Tabela 2:** – Determinantes clínicos e demográficos da regurgitação valvar mitral, aórtica e tricúspide moderada/grave

Covariáveis	Regurgitação mitral				Regurgitação aórtica				Regurgitação tricúspide			
	Modelo 1 (ajustado por idade/sexo)		Modelo 2*		Modelo 1 (ajustado por idade/sexo)		Modelo 2*		Modelo 1 (ajustado por idade/sexo)		Modelo 2*	
	OR (IC95%)	Valor p	OR (IC95%)	Valor p	OR (IC95%)	Valor p	OR (IC95%)	Valor p	OR (IC95%)	Valor p	OR (IC95%)	Valor p
Idade (anos)	1,07 (1,02-1,13)	<0,01	1,07 (1,01-1,13)	0,02	1,12 (1,04-1,20)	<0,01	1,10 (1,03-1,19)	<0,01	1,03 (0,96-1,09)	0,36	1,04 (0,97-1,11)	0,26
Sexo masculino	0,83 (0,42-1,65)	0,61	0,80 (0,40-1,61)	0,54	1,18 (0,79-4,14)	0,15	1,82 (0,79-4,21)	0,16	0,15 (0,03-0,69)	0,01	0,18 (0,14-0,82)	0,02
Raça (preta)			3,28 (1,58-6,82)	<0,01			2,41 (0,97-5,98)	0,06			3,70 (1,29-10,55)	0,01
Tabagismo			1,23 (0,42-3,57)	0,70			1,29 (0,38-4,45)	0,68			0,69 (0,09-5,31)	0,72
Obesidade			0,27 (0,08-0,89)	0,03			0,48 (0,41-1,62)	0,23			1,12 (0,35-3,58)	0,85
Hipertensão			1,57 (0,71-3,49)	0,26			5,48 (1,59-18,9)	<0,01			1,21 (0,41-3,53)	0,72
Diabetes			1,49 (0,71-3,11)	0,29			0,34 (0,11-1,02)	0,06			0,16 (0,02-1,23)	0,08

*Ajustado por idade, sexo, tabagismo, hipertensão e diabetes.

Na regressão logística multivariada, RM moderada/grave foi independentemente associada à idade mais avançada, raça preta e obesidade. RA moderada/grave esteve associada à idade mais avançada e hipertensão. RT moderada/grave foi associada ao sexo feminino e à raça preta (Tabela 2).

Como esperado, o grupo com DVC moderada/grave apresentou maiores dimensões atriais e ventriculares esquerdas, maior massa ventricular esquerda, menor espessura relativa da parede e menor fração de ejeção do VE, além de maior prevalência de hipertensão pulmonar em comparação aos demais grupos (Tabela 1).

Pequenas diferenças nos padrões de remodelamento foram identificadas entre os fenótipos de DVC moderada/grave mais prevalentes (RM, RA e RT), mesmo após ajuste por idade e sexo (Material Suplementar – Figura 3).

**Figura 2:**
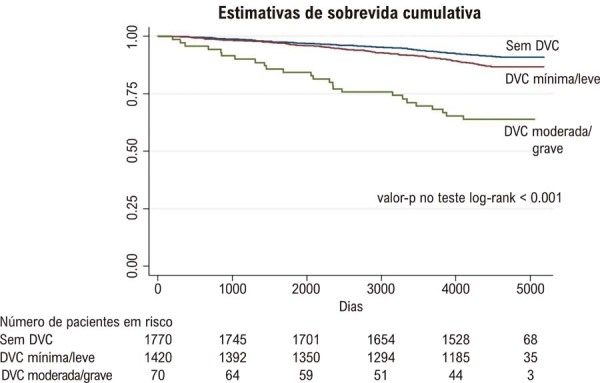
– Curvas de sobrevida de Kaplan–Meier dos participantes incluídos no ELSA, de acordo com a gravidade da doença valvar cardíaca (DVC).

Ao todo, 355 participantes (10,9%) morreram durante um seguimento médio de 4.260 ± 840 dias. Os participantes com DVC moderada/grave apresentaram pior sobrevida, conforme demonstrado na curva de Kaplan–Meier (log-rank p < 0.001; Figura 2 e Ilustração Central). A DVC moderada/grave foi identificada como preditor independente de mortalidade (HR ajustado: 3,7; IC 95%: 2,17–6,31), após ajuste por idade, sexo e hipertensão.

## Discussão

Em uma população latino-americana de meia-idade, observamos que a DVC é mais prevalente entre indivíduos mais velhos. Participantes com DVC moderada/grave apresentaram maior carga de fatores de risco cardiovascular, remodelamento cardíaco mais acentuado, menor fração de ejeção do VE e pior sobrevida em comparação àqueles sem doença valvar significativa.

Em nossa população, a prevalência de DVC moderada/grave foi semelhante à relatada em uma coorte hispânica/latina dos Estados Unidos e em um estudo comunitário recente envolvendo idosos assintomáticos.^1,3^ De forma consistente com estudos anteriores, encontramos associação independente entre idade e todos os tipos de regurgitação valvar moderada/grave.^1,5,13^ No entanto, estudos com maior proporção de idosos relataram prevalência mais elevada de DVC moderada/grave do que a observada em nossa coorte.^5,7,14^ O estresse hemodinâmico progressivo imposto às valvas ao longo dos anos provavelmente contribui para alterações degenerativas, sustentando o aumento da prevalência de DVC com o avançar da idade. Entre as diversas lesões, a regurgitação mitral foi a mais frequente, em concordância com a maioria dos estudos populacionais previamente publicados.^1-3,7^ A estenose aórtica também é uma doença valvar altamente prevalente, mas sua frequência aumenta substancialmente com o envelhecimento populacional.^5,15-19^ Em nossa amostra, observamos maior prevalência de regurgitação aórtica do que de estenose aórtica, possivelmente refletindo a idade média relativamente mais baixa da coorte (60 anos).

A coorte ELSA-Brasil, que inclui participantes de seis estados distribuídos por três grandes regiões do país, captura a diversidade socioeconômica, racial e climática do Brasil. É notável que 44% de nossa amostra era composta por participantes não brancos, e indivíduos que se autodeclararam pretos apresentaram maiores taxas de RM e RT moderadas/graves. Não foi possível determinar se fatores genéticos, determinantes sociais ou diferenças no acesso à saúde contribuíram para esses achados. Ainda assim, ao incluir um grupo populacional historicamente sub-representado em pesquisas, nosso estudo reforça a necessidade de investigações mais amplas e inclusivas.

Assim como em outros estudos, observamos que o grupo com DVC moderada/grave apresentou maior prevalência de doenças cardiovasculares.^2,3,7^ Notavelmente, a hipertensão foi independentemente associada à regurgitação aórtica, em consonância com relatos que sugerem que a hipertensão pode predispor ao aumento da raiz aórtica e, consequentemente, à regurgitação aórtica.^20^

Observamos câmaras cardíacas maiores no grupo com DVC moderada/grave, consistentes com o remodelamento ventricular e atrial que ocorre em resposta à sobrecarga hemodinâmica imposta pela DVC. A maioria dos estudos anteriores também relatou maiores dimensões atriais e ventriculares esquerdas, maior massa ventricular esquerda, menor fração de ejeção do VE e hipertensão pulmonar em participantes com DVC.^1,5,7^ Esse fenótipo foi particularmente evidente na RM moderada/grave, o que pode refletir as consequências da sobrecarga crônica de volume e do aumento do estresse parietal sobre a estrutura e a função cardíacas.

Fatores socioeconômicos também podem influenciar o cenário da DVC, uma vez que atrasos e barreiras no acesso ao diagnóstico, tratamento e prevenção podem impactar de forma crítica os desfechos da doença. Em países de renda média, como o Brasil, e em contextos sociais mais vulneráveis, pode haver maior prevalência de DVC e maior probabilidade de desfechos desfavoráveis.

Alterações estruturais e funcionais cardíacas associadas à DVC podem contribuir para piores desfechos clínicos e podem explicar a menor sobrevida observada nos participantes com DVC moderada/grave em nosso estudo, assim como em pesquisas anteriores.^5,21^ Diante do envelhecimento populacional global e da forte associação entre DVC moderada/grave e idade avançada, compreender a progressão e a mortalidade associadas à DVC torna-se cada vez mais importante.

Não conseguimos identificar um padrão ecocardiográfico específico típico da doença cardíaca reumática nessa população de meia-idade. Em indivíduos mais jovens, a presença de regurgitação valvar combinada ou doença valvar mista é um marcador importante de envolvimento reumático e é amplamente utilizada em programas de rastreamento em larga escala. No entanto, em populações mais idosas, a especificidade desses achados é limitada, pois etiologias degenerativas e funcionais tornam-se mais prevalentes e acabam predominando sobre os padrões reumáticos.^22^

Nosso estudo apresenta algumas limitações. A coorte foi composta por servidores públicos de universidades e instituições de pesquisa, o que pode não representar completamente a população brasileira geral. Por se tratar de uma coorte ocupacional, é possível que indivíduos com doenças mais incapacitantes não tenham sido incluídos. A faixa etária relativamente estreita, com a maioria dos participantes sendo de meia-idade, pode ter levado à subestimativa de casos moderados a graves, e não foi possível determinar a etiologia das principais DVC nativas. Além disso, o delineamento transversal impede avaliar como a DVC influencia desfechos de longo prazo, incluindo sua contribuição direta para a mortalidade. Ademais, o estudo foi conduzido com base em um protocolo pré-definido, e as avaliações ecocardiográficas foram qualitativas, sem quantificação detalhada de lesões valvares específicas.

Nesta coorte brasileira de meia-idade, a DVC mostrou-se prevalente e aumentou com o avanço da idade. Participantes com DVC moderada/grave apresentaram maior carga de comorbidades, remodelamento cardíaco mais acentuado, pior função sistólica e menor sobrevida. Compreender a DVC no contexto brasileiro atual pode contribuir para aprimorar o planejamento em saúde pública e favorecer o diagnóstico precoce e o manejo oportuno da doença.

## Material suplementar

Supplementary material
