# Supplementary material for: Epidemiologia e Mortalidade da Doença Valvar Cardíaca em Adultos Brasileiros: Achados da Coorte ELSA-Brasil
Source: Arq Bras Cardiol. 2026 Jun 26;123(6):e20260060. [Article in Portuguese] doi: 10.36660/abc.20260060 (PMC13399093; doi:10.36660/abc.20260060)
Supplement: Supplementary material [file 0066-782x-abc-123-6-e20260060-suppl01.pdf]

Supplementary material

Table 1 - Number of participants with VHD in each age group

	35-44 y<br>n=224	45-54y<br>n=510	55-64y<br>n=1,360	65-75y<br>n=1,166	p-value
**AR**	5 (2.2)	23 (4.5)	170 (12.5)	279 (23.9)	<0.01
**AS**	0 (0)	0 (0)	3 (0.2)	13 (1.1)	<0.01
**MR**	28 (12.5)	74 (14.5)	329 (24.2)	398 (34.1)	<0.01
**MS**	0 (0)	1 (0.2)	1 (0.1)	4 (0.3)	0.22
**TR**	33 (14.7)	91 (17.8)	378 (27.8)	449 (38.5)	<0.01

Values are shown as N (%); p from trend in age strata
AR: aortic regurgitation; AS: aortic stenosis; MR: mitral regurgitation; MS: mitral stenosis TR: tricuspid regurgitation.

Table 2 – Univariate analysis of moderate/severe mitral regurgitation

	Univariate		
	**Odds Ratio**	**95%CI**	**p-value**
**Age**	1.07	1.02 – 1.13	<0.01
**Sex-Male**	0.88	0.44 – 1.75	0.73
**Race Categorical-Black**	3.5	1.74 – 7.06	<0.01
**Brazilian centers**	0.93	0.76 –1.13	0.48
**BMI**	0.93	0.85 – 1.01	0.18
**Weight**	0.98	0.96 – 1.0	0.22
**Height**	0.88	0.024 – 32.08	0.94
**BSA**	0.45	0.08 – 2.5	0.36
**SBP**	1.05	0.98 – 1.02	0.55
**DBP**	0.97	0.94 – 1.00	0.13
**Obesity**	0.33	0.10 – 1.10	0.07
**Overweight**	1.3	0.68 – 2.63	0.39
**Smoking**	1.17	0.41 – 3.35	0.76
**Hypertension**	2.31	1.10 – 4.86	0.02
**Diabetes**	2.05	1.02 – 4.11	0.04
**Heart failure**	10.35	4.56 – 23.47	<0.01
**Coronary Heart Disease**	2.59	0.99 – 6.8	0.05
**Metabolic Syndrome**	1.18	0.59 – 2.33	0.63
**AF and/or flutter**	18.34	5.97 – 56.27	<0.01
**LV Relative Wall Thickness**	0.96	0.85 – 1.08	0.52
**LV mass, g**	1.01	1.0 – 1.01	<0.01
**LV diastolic volume, mL**	1.04	1.03 – 1.05	<0.01
**LV systolic volume, mL**	1.05	1.04 – 1.07	<0.01
**LV ejection fraction, %**	0.89	0.87 – 0.91	<0.01
**LA volume index, mL/m²**	1.11	1.08 – 1.13	<0.01
**Pulmonary hypertension**	4.29	2.07 – 8.89	<0.01

AF: atrial fibrillation; BMI: body-mass index; BSA: body surface area; DBP: Diastolic blood pressure; SBP: systolic blood pressure.

Table 3 - Univariate analysis of moderate/severe aortic regurgitation

	Univariate		
	**Odds Ratio**	**CI**	**p-value**
**Age**	1.12	1.04 – 1.20	<0.01
**Sex-Male**	2.01	0.88 – 4.56	0.09
**Race Categorical-Black**	2.18	0.91 – 5.26	0.08
**Brazilian centers**	1.11	0.88 – 1.41	0.37
**BMI**	0.93	0.84 – 1.02	0.16
**Weight**	0.98	0.95 – 1.01	0.19
**Height**	0.83	0.01 – 54.56	0.93
**BSA**	0.33	0.04 – 2.55	0.29
**SBP**	1.03	1.01 – 1.05	<0.01
**DBP**	0.96	0.92 –1.00	0.06
**Obesity**	0.47	0.14 – 1.59	0.23
**Overweight**	0.62	0.27 – 1.45	0.27
**Smoking**	1.2	0.35 – 4.03	0.76
**Hypertension**	7.10	2.12 – 23.8	<0.01
**Diabetes**	0.62	0.21 – 1.82	0.39
**Heart failure**	4.35	1.23 – 14.8	0.02
**Coronary heart disease**	4.63	1.83 – 11.72	0.01
**Metabolic Syndrome**	0.52	0.22 – 1.18	0.11
**AF and/or flutter**	5.08	0.66 – 38.96	0.11
**LV Relative Wall Thickness**	0.03	0.00 – 13.9	0.27
**LV mass, g**	1.01	1.01 – 1.02	<0.01
**LV diastolic volume, mL**	1.04	1.03 – 1.05	<0.01
**LV systolic volume, mL**	1.03	1.01 – 1.05	<0.01
**LV ejection fraction, %**	0.99	0.94 – 1.04	0.81
**LA volume index, mL/m²**	1.08	1.05 – 1.11	<0.01
**Pulmonary hypertension**	1.67	0.57 – 4.91	0.34

AF: atrial fibrillation; BMI: body-mass index; BSA: body surface area; DBP: Diastolic blood pressure; SBP: systolic blood pressure.

Table 4 - Univariate analysis of moderate/severe tricuspid regurgitation

	Univariate		
	**Odds Ratio**	**CI Ratio**	**p-value**
**Age**	1.02	0.96 – 1.09	0.42
**Sex-Male**	0.15	0.03 – 0.70	0.01
**Race Categorical-Black**	3.37	1.22 – 9.32	0.02
**Brazilian centers**	1.09	0.82 – 1.48	0.53
**BMI**	0.96	0.85 – 1.08	0.52
**Weight**	0.96	0.92 – 1.0	0.06
**Height**	0.89	0.02 - 32.1	0.95
**BSA**	0.04	0.00 – 0.64	0.02
**SBP**	0.99	0.97 – 1.02	0.85
**DBP**	0.96	0.92 – 1.01	0.19
**Obesity**	1.17	0.37 – 3.64	0.78
**Overweight**	0.44	0.14 – 1.38	0.16
**Smoking**	0.58	0.08 – 4.44	0.60
**Hypertension**	1.23	0.45 – 3.32	0.68
**Diabetes**	0.22	0.03 – 1.65	0.14
**Heart failure**	7.39	2.07 – 26.38	<0.01
**Coronary heart disease**	1.02	0.13 – 7.82	0.98
**Metabolic Syndrome**	0.21	0.06 – 0.75	0.01
**AF and/or flutter**	18.1	3.92 – 84.16	<0.01
**LV Relative Wall Thickness**	0.11	0.0 – 124.7	0.54
**LV mass, g**	0.99	0.98 – 1.00	0.46
**LV diastolic volume, mL**	0.99	0.97 – 1.02	0.86
**LV systolic volume, mL**	1.01	0.98 – 1.05	0.35
**LV ejection fraction, %**	0.95	0.90 – 1.01	0.16
**LA volume index, mL/m²**	1.07	1.04 – 1.10	<0.01
**Pulmonary hypertension**	5.33	1.92 – 14.7	<0.01

AF:atrialfibrillation; BMI: body-mass index; BSA: body surface area; DBP: Diastolic blood pressure; SBP: systolic blood pressure.

Figure 1: Flow chart of included Elsa sample

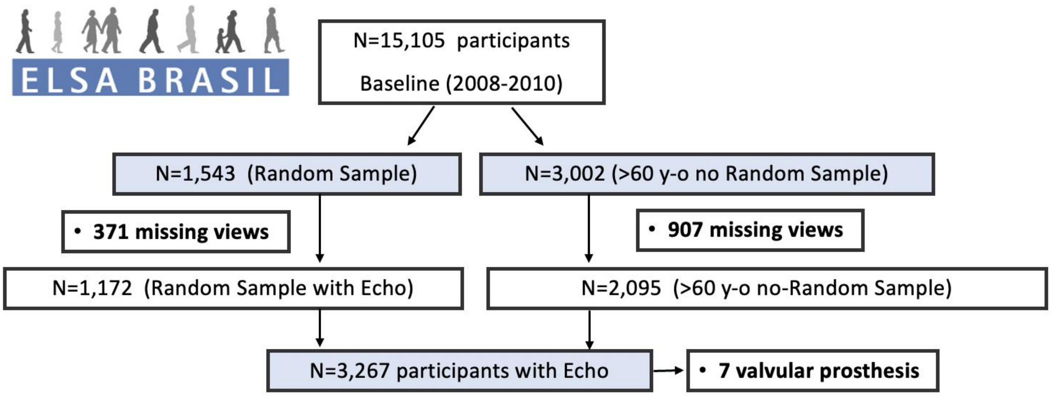

Figure 2: A Venn diagram demonstrating the prevalence of valvar regurgitation.

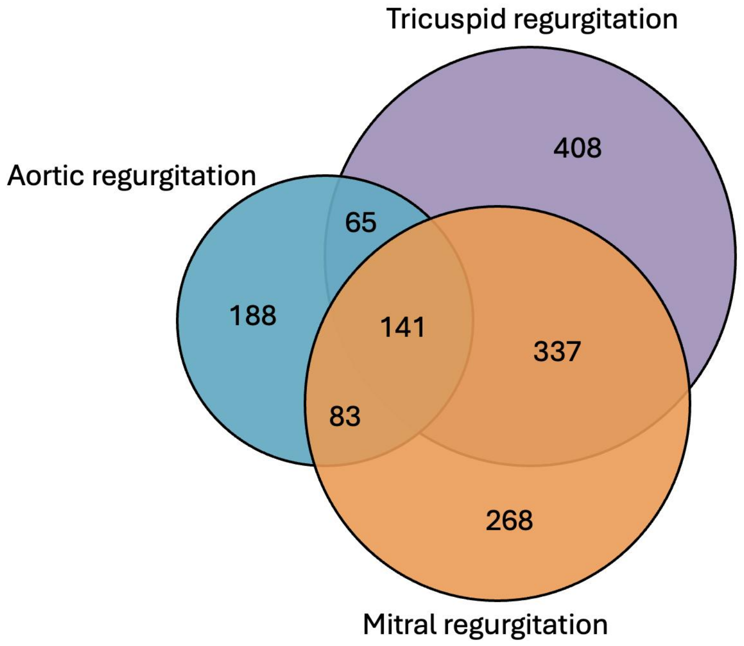

Figure 3: Echocardiographic phenotype of moderate/severe mitral, tricuspid, and aortic regurgitation.

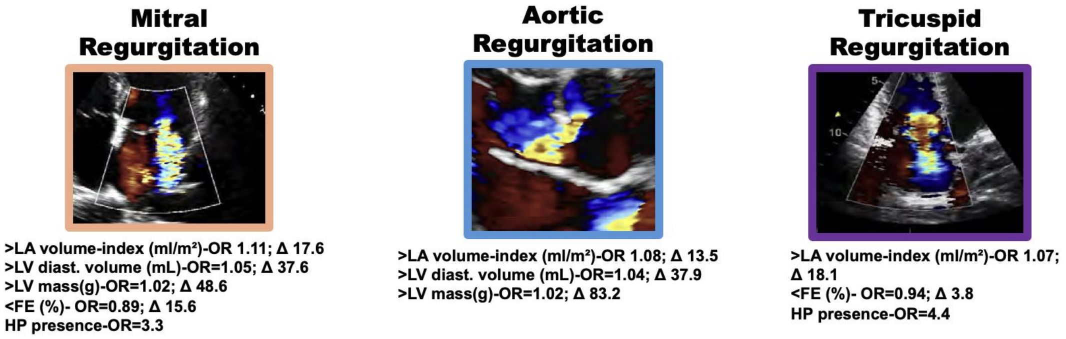

Adjusted model for age and sex: LV diastolic volume; LV mass, LV ejection fraction, LA volume atrial index, and PH.
LA: left atrial; LV: left ventricle; HP: hypertension pulmonary.
