# Supplementary material for: Epidemiologia e Mortalidade da Doença Valvar Cardíaca em Adultos Brasileiros: Achados da Coorte ELSA-Brasil
Source: Arq Bras Cardiol. 2026 Jun 26;123(6):e20260060. [Article in Portuguese] doi: 10.36660/abc.20260060 (PMC13399093; doi:10.36660/abc.20260060)
Supplement: Translation

# Translation: Epidemiology and Mortality of Valvular Heart Disease in Brazilian Adults: Findings from the ELSA-Brasil Cohort

**DOI:** 10.36660/abc.20260060i

**Authors:** Guilia Bevilacqua Schmitz, Eduardo Gatti Pianca, Maria Do Carmo Pereira Nunes, Bruce Bartholow Duncan, Murilo Foppa, Angela Barreto Santiago Santos

**Affiliations:** 1 Brazil Pós-Graduação em Cardiologia e Ciências Cardiovasculares – Faculdade de Medicina – Universidade Federal do Rio Grande do Sul, Porto Alegre, RS – Brazil; 2 Brazil Serviço de Cardiologia, Hospital de Clínicas de Porto Alegre, Porto Alegre, RS – Brazil; 3 Brazil Faculdade de Medicina, Hospital das Clínicas, Universidade Federal de Minas Gerais, Belo Horizonte, MG – Brazil; 4 Brazil Pós-Graduação em Epidemiologia, Universidade Federal do Rio Grande do Sul, Porto Alegre, RS – Brazil

**Keywords:** Heart Valve Diseases, Epidemiology, Mortality, Adult

## Abstract

**Background:**

Most of the current evidence on valvular heart disease (VHD) comes from high-income countries. Access to treatment and the impact of VHD on quality of life may vary substantially across nations, largely due to socioeconomic differences.

**Objective:**

This study aimed to estimate the burden of VHD and its association with mortality in a middle-income country.

**Methods:**

We included participants from the ELSA-Brasil cohort who underwent transthoracic echocardiography at baseline (3,267 participants; 1,539 males; mean age 60.6 ± 8.8 years) across six Brazilian states. Participants were categorized into three groups—no VHD, trace/mild VHD, and moderate/severe VHD—and their characteristics and associated factors were analyzed. P-values < 0.05 were considered statistically significant.

**Results:**

Among all participants, 2.4% had moderate/severe VHD (including 7 participants with prosthetic valves), and 43.6% had trace/mild VHD. The most prevalent moderate/severe VHD was mitral regurgitation, followed by aortic and tricuspid regurgitation, with 56 participants (86%) presenting isolated valvular regurgitation. Moderate/severe VHD was more common in older individuals and was associated with a higher prevalence of most cardiovascular comorbidities, but with lower obesity rates. It was also associated with greater cardiac remodeling and reduced systolic function, with phenotypic variations depending on the affected valve. Moderate/severe VHD was linked to increased 10-year mortality (HR: 3.7; 95% CI [2.17–6.31]), independent of age, sex, and hypertension.

**Conclusion:**

VHD is common among community-dwelling adults in this middle-income country and increases with age. Participants with moderate/severe VHD exhibited a greater burden of comorbidities, more pronounced cardiac remodeling, worse systolic function, and substantially reduced long-term survival.

## Introduction

Valvular heart disease (VHD) is a prevalent cardiac condition associated with increased morbidity and mortality,^1-3^ and its prevalence rises with age.^4-6^ Although most patients have mild and asymptomatic VHD, those with moderate or severe disease may experience significant complications, including heart failure and death.^2,3^ Moreover, the impact of VHD on quality of life and access to treatment varies according to the socioeconomic context of each country.

Several factors have been previously associated with different types of VHD. Mitral regurgitation is linked to older age, female sex, hypertension, reduced left ventricular (LV) ejection fraction (EF), and lower body mass index (BMI).^2,5,7^ Tricuspid regurgitation may be associated with older age, female sex, lower BMI, alcohol consumption, higher HDL-cholesterol, increased heart rate, LV dysfunction, left-sided heart disease, and left atrial enlargement.^6-8^ Aortic regurgitation is primarily associated with older age, male sex, higher systolic blood pressure, aortic root dilation, and increased LV mass index.

Most available VHD data originates from high-income countries, and population-based evidence from Latin America remains scarce, limiting accurate estimates of the VHD burden in this region. The ELSA-Brasil cohort provides a unique opportunity to obtain reliable estimates of the prevalence and outcomes of VHD and to assess the impact of the ongoing epidemiologic transition in a middle-income country. Therefore, the present study aimed to investigate: 1) the prevalence of VHD in a free-living Brazilian adult population; 2) the clinical and demographic determinants of VHD; 3) the echocardiographic phenotype of each moderate/severe VHD; and 4) the impact of moderate/severe VHD on long-term mortality.

## Material and Methods

### Study population

The Brazilian Longitudinal Study of Adult Health (ELSA-Brasil) is a prospective epidemiological study designed to investigate cardiovascular disease and diabetes in 15,105 male and female civil servants from universities or research institutions located in six Brazilian cities (São Paulo, Rio de Janeiro, Belo Horizonte, Vitória, Salvador, and Porto Alegre). All active or retired employees aged 35 to 74 years were eligible for participation. The study details – including design, eligibility criteria, recruitment, and assessments – have been described elsewhere.^9,10^

A cohort random subsample was defined *a priori* for case-cohort analyses. This random sample (n = 1,543) consisted of 10% of the total cohort, selected using the same stratification criteria applied to the full sample (sex, age group, and occupational category). A computer-generated random number was assigned to each participant, and the 10% lowest numbers within each stratum were selected. From this random sample, transthoracic echocardiograms (TTE) were obtained during the first study visit in 1,172 participants.

For the present analysis, we included all cohort participants who underwent baseline TTE (August 2008 to December 2010), prioritizing all individuals aged 60 years or older (N = 2,095), as well as the predefined random subsample comprising 10% of the entire cohort (N = 1,172) (Supplemental Material – Figure 1).

All-cause mortality data up to December 2022 were collected through annual telephone follow-up and confirmed by medical chart review, death certificates, and linkage to national databases such as the National Mortality System.

The ELSA-Brasil study complies with the principles of the Declaration of Helsinki and was approved by the research and ethics committees of the participating institutions. All participants provided written informed consent.

### Echocardiography

All transthoracic echocardiograms were obtained during the first study visit. Images were acquired using identically configured echocardiography machines (Aplio XG, Toshiba, Japan) equipped with a 2.5-MHz sector transducer, following standardized protocols. Examinations were recorded in digital format and transferred to the ELSA-Brasil Echocardiography Reading Center in Porto Alegre, Brazil. Standard echocardiographic and Doppler parameters were analyzed using an offline workstation (ComPACS 10.5; Medimatic SrL, Italy). All measurements were performed in triplicate according to the recommendations of the American Society of Echocardiography^1 1^ and included LV diameters and volumes, LV wall thickness, LV mass, LV ejection fraction, and left atrial (LA) volume and diameter.

VHD severity was initially flagged by the trained echocardiographer during image acquisition, and additional images beyond the pre-acquisition protocol were obtained at the sonographer’s discretion to better characterize VHD, including assessment of mitral valve prolapse and bicuspid aortic valve morphology. All images were subsequently reviewed at the reading center (regardless of initial flagging) and qualitatively assessed using color Doppler and spectral Doppler findings, following criteria applied in previous population-based studies.^12^

Additionally, the definition of aortic stenosis (AS) was based on the antegrade systolic velocity across the aortic valve, while the classification of mitral stenosis (MS) was based on the mean transmitral gradient.

The study protocol specifically aimed to identify mitral valve prolapse, defined as maximal leaflet thickness >5 mm and systolic displacement >2 mm beyond the annular plane in the parasternal view, as well as bicuspid aortic valve morphology, characterized by eccentric leaflet coaptation, the presence of two leaflets during systole, and an elliptical systolic orifice. Pulmonary hypertension was defined as a right atrial–right ventricular (RA–RV) gradient >25 mmHg.

### Statistical analysis

We categorized participants into three groups: no VHD, trace/mild VHD, and moderate/severe VHD. Continuous variables are presented as median and interquartile range and were compared using the Kruskal–Wallis test. When the global p-value was <0.05, post hoc pairwise comparisons were performed using Dunn’s test with Bonferroni correction for multiple comparisons. Categorical variables are presented as counts and proportions and were compared using Fisher’s exact test, followed by pairwise Fisher’s exact tests when the global p-value was <0.05, with p-values adjusted using the Bonferroni method.

Each valvular heart disease was analyzed across age decades using Cuzick’s non-parametric test for trend (nptrend). Relevant correlates of the most prevalent moderate/severe VHD – mitral regurgitation (MR), aortic regurgitation (AR), and tricuspid regurgitation (TR) – were first explored in univariate analyses, followed by multivariable logistic regression models including covariates selected a priori based on established literature, representing clinically distinct risk factors for valvular disease (age, sex, Black race category, smoking, obesity, hypertension, and diabetes). Additionally, we evaluated the main echocardiographic parameters associated with specific VHD phenotypes (LV end-diastolic volume, LV end-systolic volume, LV relative wall thickness, LV mass, LV ejection fraction, LA volume index, and pulmonary hypertension) using logistic regression models adjusted for age and sex.

Cumulative survival was estimated using the Kaplan–Meier method, and survival curves were compared using the log-rank test. Participants with prosthetic valves were included in the overall prevalence of moderate/severe VHD but were excluded from subsequent characterization analyses due to alterations in the natural history of VHD.

All statistical analyses were performed using the STATA software package (version 12; StataCorp, College Station, Texas). All tests were two-sided, and p-values <0.05 were considered statistically significant.

## Results

Our sample consisted of 3,267 participants (1,539 males; mean age 60.6 ± 8.8 years; 56% self-reported as White) from six different states in Brazil (15% from the Northeast, 63% from the Southeast, and 22% from the South). The prevalence of hypertension was 51.0%, diabetes 23.4%, overweight 42.8%, obesity 22.2%, coronary artery disease 6.5%, and smoking 10.2% in the overall population.

Among all participants, 2.4% had moderate/severe VHD (including 7 participants with prosthetic valves), and 43.6% had trace/mild VHD (Figure 1 and Central Illustration). The most prevalent moderate/severe VHD was mitral regurgitation, followed by aortic and tricuspid regurgitation. Among those with moderate/severe VHD, 56 (86%) had isolated valvular regurgitation; 8 (12.5%) had mixed disease involving two regurgitant valves; and 1 (1.5%) had mixed disease involving three regurgitant valves. Moderate/severe stenosis was present in 6 participants (8.5% of those with moderate/severe VHD). Additionally, the prevalence of mitral valve prolapse was 1.2%, and bicuspid aortic valve was 0.5%, with no significant differences between sexes.

**Figure 1:** – Prevalence of valvular heart disease (VHD) in the sample.

Participants older than 55 years had a higher prevalence of both trace/mild and moderate/severe VHD compared with younger individuals. Only four cases of moderate/severe VHD were identified among participants younger than 55 years, corresponding to a prevalence below 1% (Supplemental Material – Table 1).

Participants with moderate/severe VHD were older and had a higher prevalence of hypertension, coronary artery disease, heart failure, smoking, and atrial flutter/fibrillation, as well as a lower prevalence of obesity and metabolic syndrome (Table 1).

**Table 1:** – Baseline demographic characteristics and echocardiographic parameters of ELSA-Brasil participants who underwent echocardiography at baseline, stratified by valvular heart disease severity

	No VHD (n=1,770)	Trace/Mild VHD(n=1,420)	Mod/severe VHD (n=70)	p-value
**Age years**	61 (51–65)	64 (60-68)	65 (61-71)*^†^	<0.01
**Sex**				
Female	909 (51.3)	779 (54.9)	38 (54.3)	0.13
Male	861(48.6)	641 (45.14)	32 (45.7)	0.13
**Self-identified skin color/ race category**				0.005
White	993 (55.0)	783 (43.4)	29 (1.6)	
Black	243 (48.9)	231 (46.6)	22 (4.4)	
Brown (‘pardo’)	442 (55.0)	346 (43.0)	16 (2.0)	
Asian	44 (55.0)	35 (43.8)	1 (1.2)	
Indigenous	26 (68.4)	11 (29.0)	1 (2.6)	
**Brazilian centers**				<0.01
Bahia	218(44.1)	261(52.8)	15 (3.1)	
Espírito Santo	158(56.4)	119(42.5)	2 (1.1)	
Minas Gerais	390(47.5)	411(50.1)	20 (2.4)	
Rio de Janeiro	238(75.8)	73(23.2)	3 (1.0)	
São Paulo	377(58.9)	246(38.4)	17 (2.7)	
Rio Grande do Sul	389(54.7)	310(43.6)	12 (1.7)	
**SBP (mmHg)**	122.5 (112.5-134)	125 (114-139)	132 (118-142)*	<0.01
**DBP (mmHg)**	76 (69.5-83)	75.5 (69-82.5)	71.8 (65.5-80)*^†^	<0.01
**BMI (Kg/m^2^)**	26.9 (24.3-29.9)	26 (23.6-28.9)	25.4 (23.3-28.3)*	<0.01
**Overweight**	769 (43.5)	597 (42)	28 (40)	0.66
**Obesity**	437 (24.7)	275 (19.4)	10 (14.3)*	<0.01
**Hypertension**	852 (48.2)	760 (53.6)	49 (70)*^†^	<0.01
**Diabetes**	408 (23.1)	338 (23.8)	15 (21.4)	0.85
**Metabolic Syndrome**	944 (53.6)	710 (50.1)	29 (41.4)	0.03
**Stroke**	33 (1.9)	29 (2.0)	3 (4.3)	0.27
**CHD**	94 (5.5)	101 (7.3)	12 (17.4)*^†^	<0.01
**Heart failure**	36 (2)	51 (3.6)	14 (20)*^†^	<0.01
**Smoking**	220 (12.4)	107 (7.5)	6 (8.6)	<0.01
**AF and/or flutter**	6 (0.3)	17 (1.2)	4 (5.7)*^†^	<0.01
**LV diastolic diameter, mm**	45 (42-48)	45 (41-48)	50 (45-56)*^†^	<0.01
**LV systolic diameter, mm**	28 (25-30)	28 (25-31)	31 (29-40)*^†^	<0.01
**Septum thickness, mm**	10 (9-11)	10 (9-11)	10 (9-12)*	<0.01
**Posterior wall thickness, mm**	9 (8-10)	9 (8-10)	9 (8-10)	0.5
**LV Relative Wall Thickness**	0.42 (0.38-0.47)	0.42 (0.38-0.48)	0.38 (0.34-0.44)*^†^	<0.01
**LV mass, g**	138.7 (114.7-167.5)	140 (116.8-170.8)	177.8 (131.6-242.2)*^†^	<0.01
**LV diastolic volume, mL**	89.1 (77.9-101.6)	89.3 (77.3-103.9)	112.1 (92.7-142.4)*^†^	<0.01
**LV systolic volume, mL**	28.5 (23.7-34.2)	28.5 (23.3-34.7)	36.4 (30.5-59.2)*^†^	<0.01
**LV ejection fraction, %**	67.9 (62.5-72.2)	68.2 (62.8-72.9)	63.5 (52.3-68.6)*^†^	<0.01
**LA volume, mL**	45 (36.6-54.1)	47.1 (38.3-58.3)	59.8 (43.8-76.6)*^†^	<0.01
**LA volume index, mL/m^2^**	25.1 (20.8- 29.9)	27.2 (22.5- 32.6)	34.1 (25.8-45.5)*^†^	<0.01
**Pulmonary hypertension**	25 (1.4)	291 (20.5)	18 (25.7)*	<0.01

Values are shown as median and interquartile range, or n (%). p-value was calculated by Kruskal-Wallis test (post hoc analysis using Dunn’s test) or χ^2^ (post hoc analysis using χ^2^ test) with Bonferroni correction for multiple comparisons if p global <0.05. *p < 0.05 vs. no VHD, ^†^p < 0.05 vs. Trace/Mild VHD. CHD: coronary heart disease; AF: atrial fibrillation; BMI: body-mass index; DBP: diastolic blood pressure; SBP: systolic blood pressure; LV: left ventricle; LA: left atrial.

Univariable analyses of the clinical and demographic determinants of moderate/severe MR, AR, and TR are presented in the Supplemental Tables. Table 2 summarizes the results of the multivariable logistic regression analyses.

**Table 2:** – Clinical and demographic determinants of moderate/severe mitral, aortic, and tricuspid valvular regurgitations

Covariates	Mitral regurgitation				Aortic regurgitation				Tricuspid regurgitation			
	Model 1 (Adjusted by age/sex)		Model 2*		Model 1 (Adjusted by age/sex)		Model 2*		Model 1 (Adjusted by age/sex)		Model 2*	
	OR (95% CI)	p value	OR (95% CI)	p value	OR (95% CI)	p value	OR (95% CI)	p value	OR (95% CI)	p value	OR (95% CI)	p value
Age (years)	1.07 (1.02-1.13)	<0.01	1.07 (1.01-1.13)	0.02	1.12 (1.04-1.20)	<0.01	1.10 (1.03-1.19)	<0.01	1.03 (0.96-1.09)	0.36	1.04 (0.97-1.11)	0.26
Male sex	0.83 (0.42-1.65)	0.61	0.80 (0.40-1.61)	0.54	1.18 (0.79-4.14)	0.15	1.82 (0.79-4.21)	0.16	0.15 (0.03-0.69)	0.01	0.18 (0.14-0.82)	0.02
Race (Black)			3.28 (1.58-6.82)	<0.01			2.41 (0.97-5.98)	0.06			3.70 (1.29-10.55)	0.01
Smoking			1.23 (0.42-3.57)	0.70			1.29 (0.38-4.45)	0.68			0.69 (0.09-5.31)	0.72
Obesity			0.27 (0.08-0.89)	0.03			0.48 (0.41-1.62)	0.23			1.12 (0.35-3.58)	0.85
Hypertension			1.57 (0.71-3.49)	0.26			5.48 (1.59-18.9)	<0.01			1.21 (0.41-3.53)	0.72
Diabetes			1.49 (0.71-3.11)	0.29			0.34 (0.11-1.02)	0.06			0.16 (0.02-1.23)	0.08

*Adjusted for age, sex, smoking, hypertension and diabetes.

In multivariable logistic regression, moderate/severe MR was independently associated with older age, Black race, and obesity. Moderate/severe AR was associated with older age and hypertension. Moderate/severe TR was associated with female sex and Black race (Table 2).

As expected, the moderate/severe VHD group showed larger left atrial and ventricular dimensions, higher LV mass, lower relative wall thickness and LV ejection fraction, and a higher prevalence of pulmonary hypertension compared with the other groups (Table 1).

Small differences in remodeling patterns were identified across the most prevalent moderate/severe VHD phenotypes (MR, AR, and TR), even after adjustment for age and sex (Supplemental Material – Figure 3).

A total of 355 participants (10.9%) died during a mean follow-up of 4,260 ± 840 days. Participants with moderate/severe VHD had worse survival, as shown in the Kaplan–Meier curve (log-rank p < 0.001; Figure 2 and Central Illustration). Moderate/severe VHD was an independent predictor of mortality (adjusted HR: 3.7; 95% CI: 2.17–6.31), after adjustment for age, sex, and hypertension.

**Figure 2:** – Kaplan-Meier survival curves of included ELSA participants, according to valvular heart disease severity.

## Discussion

In a middle-aged Latin American population, we found that VHD is more prevalent among older individuals. Participants with moderate/severe VHD had a higher burden of cardiovascular risk factors, more pronounced cardiac remodeling, lower LV ejection fraction, and poorer survival compared with those without significant valve disease.

In our population, the prevalence of moderate/severe VHD was similar to that reported in a Hispanic/Latino cohort from the United States and in a recent community-based study of asymptomatic elderly individuals.^1,3^ Consistent with previous studies, we found that age was independently associated with all types of moderate/severe valvular regurgitation.^1,5,13^ However, studies with a higher proportion of elderly participants have reported a greater prevalence of moderate/severe VHD than observed in our cohort.^5,7,14^ The progressive hemodynamic stress imposed on cardiac valves over time likely contributes to degenerative changes, supporting the increase in VHD prevalence with advancing age. Among the various lesions, MR was the most frequent, consistent with most previously published population-based studies.^1-3,7^ Aortic stenosis is also a highly prevalent valvular disease, but its frequency increases substantially with population aging.^5,15-19^ In our sample, we observed a higher overall prevalence of aortic regurgitation than aortic stenosis, likely reflecting the relatively younger mean age of our cohort (60 years).

The ELSA-Brasil cohort, which includes participants from six states across three major regions of the country, captures Brazil’s socioeconomic, racial, and climatic diversity. Notably, 44% of our sample consisted of non-White participants, and individuals who self-identified as Black had higher rates of moderate/severe MR and TR. We were unable to determine whether genetic factors, social determinants, or differences in access to health care contributed to these findings. Nonetheless, by including a population group that has been underrepresented in prior research, our study underscores the need for broader and more inclusive investigations.

As in other studies, we found that the moderate/severe VHD group had a higher prevalence of cardiovascular diseases.^2,3,7^ Notably, hypertension was independently associated with AR, consistent with reports suggesting that hypertension may predispose individuals to aortic root enlargement and, consequently, to aortic regurgitation.^20^

We observed larger cardiac chambers in the moderate/severe VHD group, consistent with the ventricular and atrial remodeling that occurs in response to the hemodynamic burden imposed by VHD. Most previous studies have also reported larger left atrial and ventricular dimensions, higher LV mass, lower LV ejection fraction, and pulmonary hypertension in participants with VHD.^1,5,7^ This phenotype was particularly evident in moderate/severe MR, which may reflect the consequences of chronic volume overload and increased wall stress on cardiac structure and function.

Socioeconomic factors may also influence the burden of VHD, as delays and barriers in accessing diagnosis, treatment, and preventive care can critically affect disease outcomes. In middle-income countries such as Brazil, where social conditions may be more precarious, the prevalence of VHD and the likelihood of unfavorable outcomes may be higher.

Structural and functional cardiac changes associated with VHD may contribute to worse clinical outcomes and could explain the lower survival observed in participants with moderate/severe VHD in our study, as well as in previous research.^5,21^ Given global population aging and the strong association between moderate/severe VHD and advancing age, understanding the progression and mortality associated with VHD is increasingly important.

We were not able to identify a specific echocardiographic pattern typical of rheumatic heart disease within this middle-aged population. In younger individuals, the presence of combined valve regurgitation or mixed valve disease is an important marker of rheumatic involvement and is commonly used in large-scale screening. However, in older populations, the specificity of these findings is limited, as degenerative and functional etiologies become more prevalent and overshadow rheumatic patterns.^22^

Our study has several limitations. The cohort consisted of civil servants from universities and research institutions, which may not fully represent the general Brazilian population. As an occupational cohort, it is possible that individuals with more disabling illnesses were not included. The relatively narrow age range, with most participants being middle-aged, may have led to an underestimation of moderate to severe cases, and we were unable to determine the etiology of major native VHD. Additionally, the cross-sectional design prevents assessment of how VHD influences long-term outcomes, including its direct contribution to mortality. Furthermore, the study followed a predefined protocol, and echocardiographic assessments were qualitative, without detailed quantification of specific valvular lesions.

In this Brazilian middle-aged cohort, VHD was found to be prevalent and increased with age. Participants with moderate/severe VHD had a higher burden of comorbidities, more pronounced cardiac remodeling, worse systolic function, and poorer survival. Understanding VHD within the current Brazilian context may contribute to improved public health planning and earlier identification and management of the disease.

## Supplemental Materials

Supplementary material
